# Analyses of outcomes of one-stage operation for treatment of late-diagnosed developmental dislocation of the hip: 864 hips followed for 3.2 to 8.9 years

**DOI:** 10.1186/1471-2474-15-401

**Published:** 2014-11-28

**Authors:** Bo Ning, Yi Yuan, Jie Yao, Sichng Zhang, Jun Sun

**Affiliations:** Department of Pediatric Orthopaedic, Anhui Provincial Children’s Hospital, 39, Wangjiang Road, 230051 Hefei, China

**Keywords:** Developmental dislocation of the hip, One-stage operation, Age, Osteonecrosis

## Abstract

**Background:**

The one-stage procedure for treatment of older developmental dislocation of the hip (DDH) is used widely. However, the best age group for this operation is still unknown. The aims of our study were to evaluate middle-term outcomes of one-stage surgical treatment of a large number of patients with late-diagnosed DDH, and to explore the best age group for treatment.

**Methods:**

We retrospectively reviewed 652 patients with a total of 864 hip joints with DDH, all aged >18 months. All the hip joints were treated with one-stage procedures including open reduction, pelvic osteotomy, and femoral shortening. The patients were divided into three groups according to age at surgery: Group I: 1.5–2.5 years; Group II: 2.5–8 years; and Group III: >8 years. The latest clinical and radiographic outcomes, complications and avascular necrosis (AVN) of the femoral head were evaluated and compared among the three groups.

**Results:**

The mean age at surgery was 5.8 years (range: 1.5–13.2 years). The average time of follow-up was 6.2 years (range: 3.2–8.9 years). A total of 79.4% of good or excellent outcomes were obtained for clinical functional evaluation according to the McKay classification. For radiographic outcomes, 732 hips (84.7%) were classified as good or excellent according to the Severin classification. A total of 27.4% of all hips had a poor outcome according to the Kalamchi and MacEwen classification for AVN. The poorest outcomes were observed for clinical, radiographic and AVN results in Group III (*p* < 0.001). Compared with Group I, the better results for clinical and AVN outcomes were found in Group II (*p* < 0.001). However, similar clinical outcomes were observed between Groups I and II (*p* > 0.05). A significantly higher incidence of redislocation and residual acetabular dysplasia was observed in Tonnis grade II and III hip dislocation (*p* < 0.001).

**Conclusions:**

One-stage treatment of late-diagnosed DDH had a good outcome in young and middle group. Younger patients achieved better results than older patients. However, the best age group was 2.5–8 years. Tonnis grade II and III DDH is a risk factor for redislocation and residual acetabular dysplasia after the one-stage operation.

**Electronic supplementary material:**

The online version of this article (doi:10.1186/1471-2474-15-401) contains supplementary material, which is available to authorized users.

## Background

Developmental dislocation of the hip (DDH) is one of most frequent deformities in children [1–3]. The goal of treatment of DDH is to achieve a stable and painless hip [4, 5]. The radiographic goal of DDH treatment is to achieve a normal acetabular index and a reduced hip joint according to Tonnis grade [6]. Although treatment of DDH is often successful in infants, it is still a challenge in late-diagnosed DDH [7, 8]. Late diagnosis has to be avoided by ultrasound screening in technique described by Graf way [9]. Muscle contractures around the joint and soft tissue pathology hinder reduction and prevent normal development of the femoral head by inflicting pressure [4]. Long-term dislocation of the hip can lead to an augmented femoral head and reduced acetabulum. All of these factors may result in difficult reduction of DDH.

Some recent studies have shown that one-stage treatment of DDH in children of walking age is feasible [5]. In the procedure, femoral shortening can facilitate reduction and reduce the risk of osteonecrosis [10–12]. Among the various methods of pelvic osteotomy, the most widely known is that of Salter, Pemberton and Dega, which is used to redirect the true acetabulum in order to obtain femoral head coverage [13–15]. The Steel and Chiari osteotomy was used to older children [16, 17]. Many authors have reported success with a single-stage procedure consisting of open reduction, capsulorrhaphy, femoral shortening, and pelvic osteotomy [10, 12, 18, 19]. Although one-stage treatment is feasible for late-diagnosed DDH, the treatment of older children is complex, with many unpredictable problems [10, 20]. There have been good and poor radiographic outcomes in different studies.

In our hospital between 2005 and 2010, there were 864 hips in 652 children with late-diagnosed DDH who were diagnosed after 18 months of age. They received one-stage surgical treatment including open reduction, capsulorrhappy, femoral shortening, and pelvic osteotomy. The purposes of the present study were to evaluate middle-term outcomes (3.2 to 8.9 years, mean 6.2 years) of one-stage surgical treatment of late-diagnosed DDH at different ages and to explore the best age group for treatment. Because of these purposes, the age of all patients at surgery is over 18 months. The latest clinical and radiographic outcomes, complications and avascular necrosis (AVN) of the femoral head were evaluated. The risk factors for poor outcomes were also analyzed.

## Methods

The study was approved by Clinical Ethics Committee of Anhui Medical University. The informed consent for participation in the study was obtained from parents of children. We retrospectively reviewed 652 patients with a total of 864 hip joints with DDH, all aged >18 months. All hip joints were treated with one-stage open reduction and Salter, Pemberton, Dega, Chiari or Steel osteotomy, combined with femoral shortening and derotation osteotomy if necessary, by three senior surgeon from 2005 to 2010 at our hospital. Pelvic osteotomies were decided according to age, grade of dislocation and acetabular index. Femoral shortening osteotomies were performed on all hip, however derotational osteotomies were done only part of cases according to CT scan preoperatively (Table 1). All patients in the present study are diagnosed DDH treated by one-stage operation. The patients with DDH did not receive one-stage operation that was excluded. Patients with known neuromuscular, pathological dislocation of the hip such as previous hip infection and syndromic disorders were excluded.

**Table 1:** **Summary of osteotomies of DDH in the present study**

Osteotomies	Young	Middle	Older	Total
Pelvic				
Salter	83	336	10	429
Pemberton	123	211	8	342
Steel	0	29	62	91
Chiari	0	0	2	
Femoral				
Shortening	206	576	82	864
Derotation	28	432	64	524

In the 652 patients (864 hips), the mean age at surgery was 5.8 years (range: 1.5–13.2 years). Two hundred and twelve (32.5%) of the 652 patients had bilateral DDH, and 440 (67.5%) had unilateral DDH. The patients were divided into three groups according to age at surgery: Group I (young): 1.5–2.5 years; Group II (middle): 2.5–8 years; and Group III (older) : >8 years (Table 2). The degree of dislocation was determined by Tonnis grade [21]. Detailed clinical information about these patients is shown in Table 2. The operative technique was similar to that described in previous studies [7]. In brief, a classical Bikini approach was used and an iliopsoas tenotomy was routinely performed, followed by removal of the ligmentum teres and incision of the transverse acetabular ligament. Any fibrous/fatty tissue in the acetabular fossa was removed, and the limbus was preserved. Different techniques of pelvic osteotomy were applied in different patients according to our preoperative clinical evaluation, age, X-ray (acetabular index) analysis and computed tomography (CT) scan (development and rotation of the acetabulum). Chiari osteotomy was used only in two patients (aged 12.6 and 13.2 years) with poor acetabular conditions in whom central reduction was not possible. Redundant capsules were excised and capsulorrhaphy performed. Femoral shortening and derotation osteotomy (DRO) were performed at the intertrochanteric region through a separate lateral incision. The length of femoral shortening was determined by the extent of dislocation (distance from the centre of the femoral head to the H line). Hip anteversion was measured by preoperative three-dimensional CT to decide the degree of femoral DRO. Osteotomy of the proximal femur was then fixed by four holes or a 90° plate. Patients were immobilized postoperatively with 45° abduction and 30° flexion in a 1.5 hip spica for 6 weeks. Indications for osteotomy were as described previously [22]. No traction was used preoperatively.

**Table 2:** **Summary of clinical features of study subjects**

Characteristics	Young	Middle	Older	Total
Patients (hips)	183(206)	391(576)	78(82)	652(864)
Age (y)	1.9 ± 0.23	4.6 ± 0.65	9.6 ± 1.2	5.8 ± 2.12
Sex				
Male (%)	29(16.1)	57(14.7)	13(17)	99(15.2)
Female (%)	154(83.9)	334(85.3)	65(83)	553(84.8)
AI (d)	43.5 ± 5.12	41.2 ± 3.12	42.6 ± 1.5	42.2 ± 6.32
Tonnis grade (hips)				
Grade I	34	86	16	136
Grade II	56	157	46	259
Grade III	77	186	10	273
Grade IV	39	147	10	196
Follow-up (y)	6.4 ± 2.81	6.12 ± 2.23	6.15 ± 2.45	6.20 ± 2.98

The medical records and serial follow-up radiographs were reviewed. The mean time of follow-up was 6.2 years (range: 3.2–8.9 years). Clinical data including pain, gait pattern, range of hip joint motion, and status of Trendelenburg sign were evaluated for each patient at the latest follow-up, using the McKay classification [23, 24] by two senior authors. The Severin grading system [24, 25] was used to evaluate the latest radiographic outcome. AVN of the femoral head was assessed using the Kalamchi and MacEwen classification [24, 26]. Postoperative complications and subsequent surgery were observed during the follow-up period.

SPSS version 16.0 was used for data analysis. Factors associated with AVN of the femoral head and the latest McKay and Severin classifications were analyzed by multiple logistic regression models. Fisher’s exact test was used to compare continuous variables among the three groups. The *χ*^2^ or Fisher’s exact test was used to compare categorical data. A *p* value <0.05 (two-sided) was considered statistically significant.

## Results

Clinical and radiographic outcomes were followed. At the time of latest follow-up, 732 (84.7%) of the 864 hips had a satisfactory radiographic outcome (Severin class Ia, Ib, or II) (Table 3). Analysis of functional outcome at the latest clinical evaluation demonstrated that 687 (79.4%) of the 864 hips had satisfactory outcome (excellent or good) according to the McKay classification (Table 4). However, 237 (27.4%) of the 864 hips had osteonecrosis of the femoral head classified as Kalamchi class II or higher (Table 5). For other postoperative complications, 14 patients had redislocation (1.6%) and 26 residual acetabular dysplasia (3.0%) that required reoperation. A significantly higher incidence of redislocation and residual acetabular dysplasia (32 of 40 cases, 80%) was observed in patients with Tonnis grade II and III hip dislocation (*p* < 0.001) (Table 6).

**Table 3:** **Results according to Severin classification for radiographic evaluation**

Severin (%)	Young	Middle	Older	Total
Ia	94(45.6)	196(34.5)	8(9.7)	298(34.5)
Ib	36(17.5)	158(27.8)	13(15.9)	207(23.9)
II	42(20.4)	177(30.7)	8(9.7)	227(26.3)
III	25(12.1)	31(5.4)	13(15.9)	69(8.0)
IV	9(4.4)	8(1.4)	6(7.3)	23(2.7)
V	0	6(1.2)	21(25.6)	27(3.1)
VI	0	0	13(15.9)	13(1.5)

**Table 4:** **Results according to McKay classification for clinical evaluation**

McKay (%)	Young	Middle	Older	Total
Excellent	112(54.4)	362(62.8)	6(7.3)	480(55.6)
Good	62(30.1)	137(23.9)	8(9.8)	207(23.8)
Fair	28(13.6)	56(9.7)	43(52.4)	127(14.8)
Poor	4(1.9)	21(3.6)	25(30.5)	50(5.8)

**Table 5:** **Results according to Kalamchi and MacEwen for osteonecrosis evaluation**

	Kalamchi (%)	Young	Middle	Older	Total
Absent	89(43.3)	393(68.3)	12(14.7)	494(57.2)	
I	46(22.3)	82(14.2)	5(6.1)	133(15.4)	
II	38(18.4)	74(12.8)	24(29.3)	136(15.7)	
III	25(12.1)	15(2.6)	26(31.7)	66(7.6)	
IV	8(3.9)	12(2.1)	15(18.2)	35(4.1)

**Table 6:** **Correlation analysis between complications and Tonnis grade**

Tonnis	Preoperative n(%)	Redislocation	Residual acetabular dysplasia
I	136(15.7)	0	4
II	259(30.0)	8	9
III	273(31.6)	5	10
IV	196(22.7)	1	3

To see radiographic outcomes in different groups, we used the Severin classification system. Thirty-four (16.5%) of the 206 hips had a poor Severin classification (above class II) in the young group. In the middle group, poor Severin classification was observed in 45 (7%) of 576 hips. In the older group, we determined 53 (64.6%) of the 82 hips had a poor Severin classification. According to the data, the latest radiographic outcomes were significantly worse in the older group when compared with the young and middle groups (*p* < 0.001). Moreover, the middle group had better radiographic results than the younger children had (*p* < 0.01) (Table 3).

The clinical outcomes were evaluated according to McKay’s scoring system. At the end of the study, 32 (15.5%) of the 206 hips were in fair or poor condition in the young group. In the middle group, 77 (13.3%) of 576 hips were rated fair or poor. In the older group, 68 (82.9%) of 82 were rated as poor. The difference in the McKay classification between the young and middle groups was not significant. However, there was a significantly higher incidence of poor McKay classification in the older children (Table 4). A good Severin classification usually resulted in a good clinical outcome in the young and middle groups. However, in the older group, poor radiographic outcome for Chiari osteotomy showed good function of the hip. For Steel osteotomy in older children, some patients obtained a good Severin classification but poor clinical outcome.

To evaluate AVN of the femoral head postoperatively, we used the Kalamchi and MacEwen classification. In the young group, we observed poor classification in 71 (34.4%) of 206 hips. In the middle group, 101 (17.5%) of 576 hips had poor classification. Sixty-five (79.2%) of 82 hips showed a poor outcome. The older group had a significantly higher incidence of osteonecrosis of the femoral head compared with the young and middle groups. The young group had a worse outcome when compared with the middle group (Table 5).

Multiple logistic regression analysis was used to assess the risk factors for poor Severin and McKay classification and for AVN of the femoral head. The preoperative variables included age, acetabular index, sex, and Tonnis grade. Older age at surgery (*p* < 0.001) and higher Tonnis grade (*p* < 0.001) were associated with a greater severity of osteonecrosis and poorer McKay classification. The data showed that too young age at surgery was a risk factor for osteonecrosis of the femoral head. Older age at surgery (*p* < 0.001) was significantly associated with a poor Severin classification, whereas sex and preoperative acetabular index and Tonnis grade were not associated with radiographic outcome.

## Discussion

**Figure 1:**
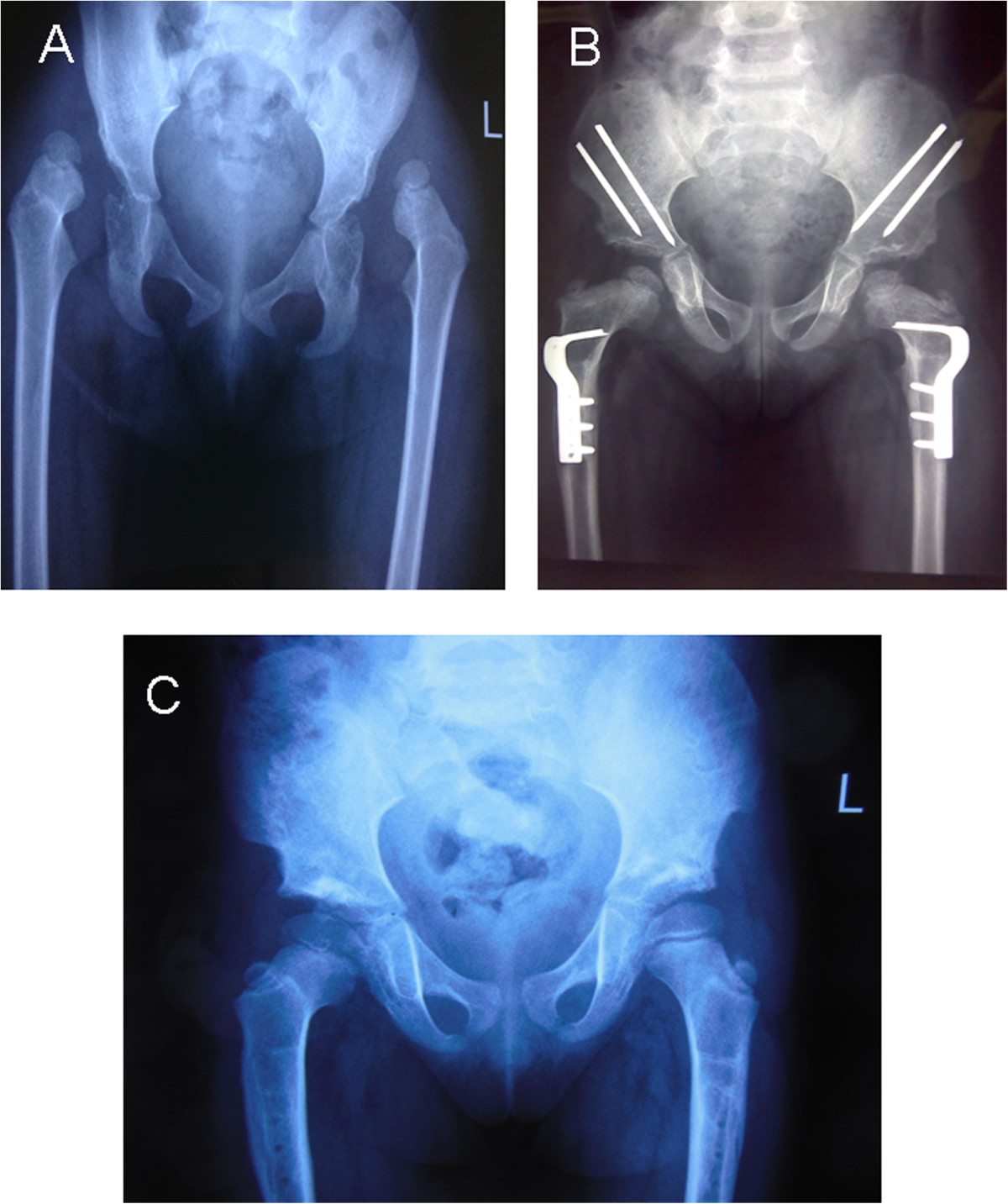
**Shows a typical X-ray in a bilateral DDH, both before operation and at the follow up examination. A**. Female patient aged 3.8 years with bilateral DDH. Plain X-ray anteroposterior (AP) view. **B**. Plain X-ray AP view 1 year after one-stage operation, showing good containment of the femoral head. **C**. AP view 3.9 years postoperatively with excellent clinical and radiographic results.

The goal of treatment of DDH is the same in infants and older children, which is concentric reduction and congruity of the femoral head into the true acetabulum [4, 5, 22, 27]. In some studies, age at surgery was an important risk factor for treatment failure and treatment of older children is still a challenge. Concentric reduction in older children may be prevented by soft tissue contracture with undue pressure on the femoral head. Acetabular dysplasia and flattened femoral head that is aggravated with increasing age, lead to difficulty of reduction. Poor hip joint motion and osteonecrosis of the femoral head may result from forced reduction. It has been reported that femoral shortening can facilitate reduction, improve joint motion, and reduce the risk of avascular necrosis of the femoral head [12, 14, 20]. It has been reported that a one-stage operation including open reduction and appropriate pelvic osteotomy may be the best option for treatment of late-diagnosed DDH [14, 19, 20, 28]. In the past 8 years, numerous older patients with DDH have been treated with a one-stage operation in our institution. We reviewed the outcomes of these patients with a minimum of 3.2 years follow-up (Figure 1). Although there were various osteotomies in the present study, most of our pelvic osteotomies were Salter and Pemberton osteotomy (Table 1). Moreover, our three seniors have the same decision tree before operation. Pelvic osteotomies were decided according to age, grade of dislocation and acetabular index. We have so many patients, the effect of various single-stage procedures were decreased to very small.

Many studies have reported the functional and radiographic outcomes of a one-stage combined operation for DDH. Most authors have achieved satisfactory results in young children. Karakas et al. [12] obtained 67% clinically and 65% radiographically good or excellent outcomes according to McKay and Severin criteria in 55 hips with a one-stage operation. Forlin et al. [29] reported on 20 children (24 hips) with untreated DDH who had a one-stage operation between the ages of 4 and 12 years. Seventeen (70%) of those hips had good or excellent results according to the McKay and Severin classifications. Meanwhile, they reported poor outcomes in patients older than 7 years at the time of surgery. Umer et al. [4] reviewed 23 patients (29 hips) who were treated with a one-stage operation. The average patient age at the time of surgery was 6.84 years. After a mean follow-up of 19.6 months, 25 (86%) hips were classified as excellent or good outcome according to the McKay classification. Ganger et al. [30] reported that 80% of hips treated with a one-stage operation had excellent or good results according to the Severin classification after a mean follow-up of 3.5 years. Galpin et al. [20] reported that 75–85% of patients had good results radiographically and clinically. All of these studies have shown that the younger the patients receive the operation, the better the results achieved. Our findings showed the similar outcomes with these studies in clinical and radiographic results. However, the best age group for a one-stage operation for DDH is still unknown.

**Figure 2:**
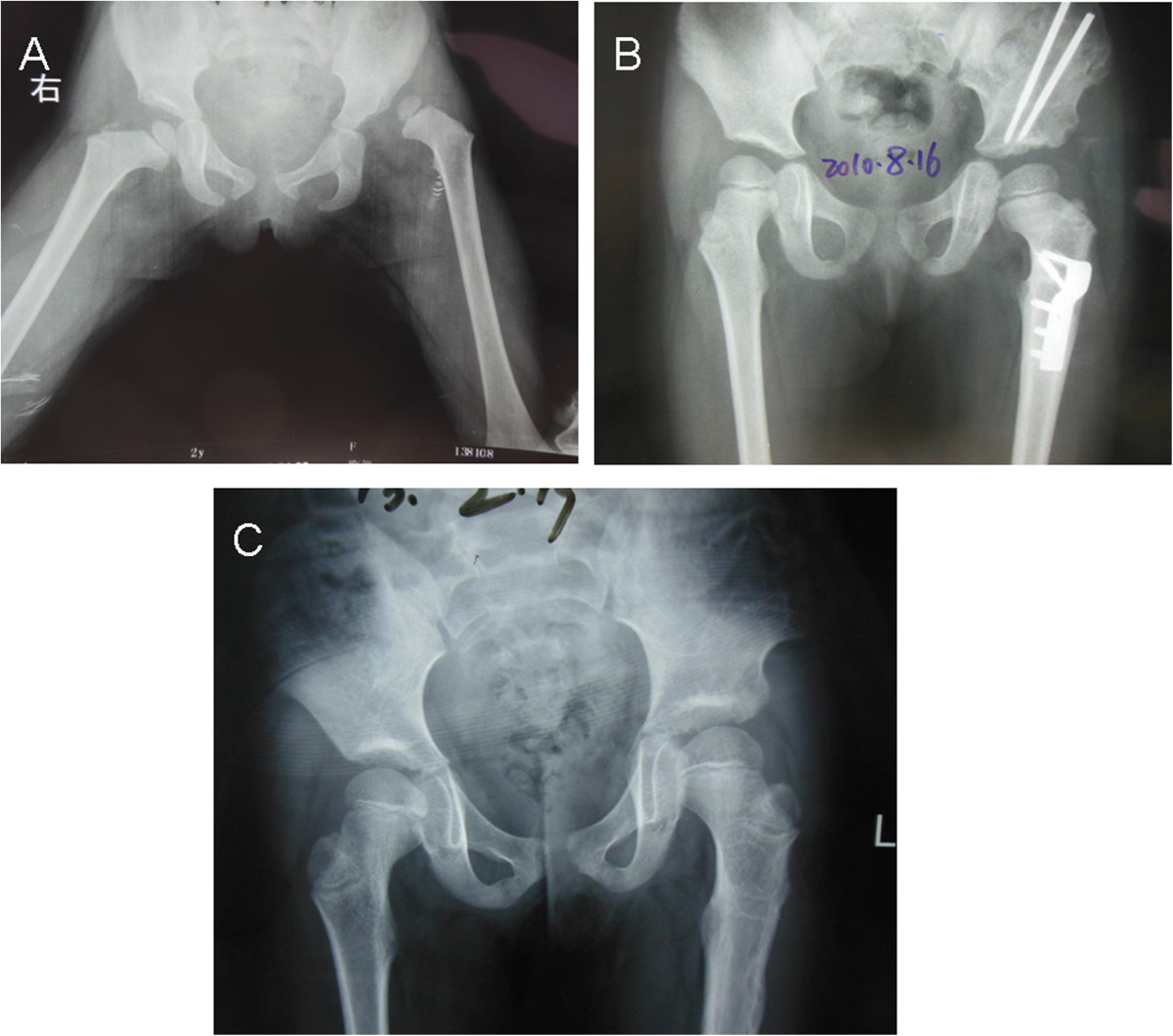
**shows X-ray in a unilateral DDH, before therapy and at the follow up examination. A**. Female patient aged 2.1 years with left DDH. Plain X-ray AP view. **B**. Plain X-ray AP view 1.6 years after one-stage operation, showing good containment of the femoral head. **C**. AP view 4.5 years postoperatively with excellent clinical and radiographic outcomes.

In the present study, we reviewed the outcomes of the one-stage operation for treatment of DDH in 864 hips in children older than 18 months. The McKay and Severin classifications were used to evaluate the clinical and radiographic results, respectively. We showed that 79.4% of good or excellent outcomes were obtained for clinical functional evaluation according to the McKay classification. For radiographic outcomes, 732 hips (84.7%) were classified as good or excellent according to the Severin classification after a mean follow up of 6.2 years. The results are similar to those in other reports [7, 29, 30]. It has been widely reported that AVN of the femoral head is still a severe complication after treatment of DDH. Our results showed that 27.4% of all hips had a poor outcome according to the Kalamchi and MacEwen classification. Previous studies have shown that sufficient femoral shortening and appropriate position of the hip spica might avoid AVN [21, 23]. In the present study, sufficient femoral shortening and stable reduction during the operation were required. After operation, 45° abduction and 30° flexion of the hip was immobilized for 6 weeks. Multiple logistic regression analysis showed that age at surgery and Tonnis grade were risk factors for AVN and poor McKay classification. For poor Severin classification, only age at surgery was a risk factor. These data support those of other researchers [7, 22]. In the present study, good Severin classification showed good clinical outcome in the young and middle groups. However, patients in the older group with poor radiographic outcome after Chiari osteotomy presented with good function of the hip. Some older patients who underwent Steel osteotomy obtained good Severin classification but poor clinical outcome. Furthermore, we failed in 21% of our cases in McKay scores, but failure analysis shows that the majorities of poor scores of McKay were in older patients. These results suggested that age at surgery was an important risk factor for clinical outcome. It is important that walking-age children with DDH should be diagnosed early and treated by one-stage operation for a satisfactory outcome (Figure 2).

Although we recommend early treatment of DDH by one-stage operation in children above walking age, the best age group at which to perform this operation is still unclear. To explore the best age group, we divided our patients into three groups according to age at surgery. In the oldest age group, patients had the poorest Severin classification for radiographic outcome among the three groups, the poorest McKay grade for clinical results, the poorest Kalamchi and MacEwen classification for osteonecrosis. Patients in the youngest age group showed poorer results than the middle group for Severin and Kalamchi and MacEwen classifications. The young and middle groups had similar results for McKay classification for clinical outcome. The finding that older patients with DDH had the poorest results supports those of other investigators. However, the best outcome in our patients was in Group II (middle age group) rather than Group I (young group). These data clearly showed that the final outcome of the one-stage operation is greatly influenced by the age of the child at the time of surgery, as assessed by radiographic and clinical analyses. Our results suggested that age at surgery played an important role in outcome of treatment of DDH with the one-stage operation. The best outcome was in Group II (2.5–8 years) rather than the youngest group. These mean that one-stage operation for DDH may be not the younger the patient performed at surgery time, the better the results was, rather to a special age group. The age group for best outcome needs further investigation.

Redislocation and residual acetabular dysplasia are inevitable complications during treatment of DDH. It has been reported that incidence of these complications after open reduction is 0–8% [11, 31, 32]. Bilateral DDH, enlarged femoral head and abnormal femoral version, and improper pelvic osteotomy may be risk factors for redislocation and residual acetabular dysplasia [7, 22, 32]. In the present study, 14 patients had redislocation (1.6%) and 26 residual acetabular dysplasia (3.0%) and required reoperation. The data were similar to previous studies [7, 31]. Data analysis showed that a significantly higher incidence of redislocation and residual acetabular dysplasia (32 of 40 cases, 80%) occurred in patients with Tonnis II and III hip dislocation. The results suggested that DDH with Tonnis II or III dislocation was a risk factor for redislocation or residual acetabular dysplasia after the one-stage operation. These complications may result from abnormal pressure of the femoral head on the lateral edge of the acetabulum in patients with Tonnis grade II or III DDH. The abnormal pressure leads to poor development of the acetabulum and predisposes the hip to redislocation or residual acetabular dysplasia after operation.

However, there were some limitations to our study. This was a retrospective study. The follow-up study was not long enough to evaluate the eventual outcome. A longer duration of follow-up would certainly be necessary to determine the latest results. As there were various osteotomies in the present study, so there was a possibility that the surgical method or the degree of dislocation could confound the effect of treatment.

We have reported the results of a one-stage operation for DDH in a large group of children aged >18 months, with an average follow-up of 6.8 years. We confirmed that the one-stage operation might be the best choice of treatment for late-diagnosed DDH. The best age group for the one-stage operation was the middle age group rather than the youngest one. To the best of our knowledge, this is the first study to explore the best age group for treatment of DDH with a one-stage operation. Additionally, we found that late-diagnosed DDH with Tonnis grade II or III dislocation was a risk factor for postoperative complications. These results suggested that the best age was 2.5–8 years for the one-stage operation for DDH.

## Conclusions

With the screening program according to Graf with early ultrasonic examination of the hips, the late diagnosis could be avoided. For late diagnosis DDH, one-stage treatment of late-diagnosed DDH had a good outcome. Our cases may add actually persuasiveness that one-stage operation is a good selection for late-diagnosed DDH. Younger patients achieved better results than older patients. However, the best age group was 2.5–8 years. Tonnis grade II and III DDH is a risk factor for redislocation and residual acetabular dysplasia after the one-stage operation.

## Authors’ original submitted files for images

Below are the links to the authors’ original submitted files for images. Authors’ original file for figure 1 Authors’ original file for figure 2

## References

[1] Stein-Zamir C, Volovik I, Rishpon S, Sabi R (2008). Developmental dysplasia of the hip: risk markers, clinical screening and outcome. Pediatr Int.

[2] Sharpe P, Mulpuri K, Chan A, Cundy PJ (2006). Differences in risk factors between early and late diagnosed developmental dysplasia of the hip. Arch Dis Child Fetal Neonatal Ed.

[3] Patel H (2001). Preventive health care, 2001 update: screening and management of developmental dysplasia of the hip in newborns. CMAJ.

[4] Umer M, Nawaz H, Kasi PM, Ahmed M, Ali SS (2007). Outcome of triple procedure in older children with developmental dysplasia of hip (DDH). J Pak Med Assoc.

[5] Wedge JH, Kelley SP (2012). Strategies to improve outcomes from operative childhood management of DDH. Orthop Clin North Am.

[6] Akman B, Ozkan K, Cift H, Akan K, Eceviz E, Eren A (2009). Treatment of Tonnis type II hip dysplasia with or without open reduction in children older than 18 months: a preliminary report. J Child Orthop.

[7] Wang TM, Wu KW, Shih SF, Huang SC, Kuo KN (2013). Outcomes of open reduction for developmental dysplasia of the hip: does bilateral dysplasia have a poorer outcome?. J Bone Joint Surg Am.

[8] Danielsson L (2000). Late-diagnosed DDH: a prospective 11-year follow-up of 71 consecutive patients (75 hips). Acta Orthop Scand.

[9] van de Sande MA, Melisie F (2012). Successful Pavlik treatment in late-diagnosed developmental dysplasia of the hip. Int Orthop.

[10] Ryan MG, Johnson LO, Quanbeck DS, Minkowitz B (1998). One-stage treatment of congenital dislocation of the hip in children three to ten years old. Functional and radiographic results. J Bone Joint Surg Am.

[11] Schoenecker PL, Strecker WB (1984). Congenital dislocation of the hip in children. Comparison of the effects of femoral shortening and of skeletal traction in treatment. J Bone Joint Surg Am.

[12] Karakas ES, Baktir A, Argun M, Turk CY (1995). One-stage treatment of congenital dislocation of the hip in older children. J Pediatr Orthop.

[13] Williamson DM, Glover SD, Benson MK (1989). Congenital dislocation of the hip presenting after the age of three years. A long-term review. J Bone Joint Surg (Br).

[14] Dogan M, Bozkurt M, Sesen H, Yildirim H (2005). One-stage treatment of congenital severely dislocated hips in older children through various acetabuloplasty techniques: 22 children followed for 1-5 years. Acta Orthop.

[15] Akgul T, Bora Goksan S, Bilgili F, Valiyev N, Hurmeydan OM (2014). Radiological results of modified Dega osteotomy in Tonnis grade 3 and 4 developmental dysplasia of the hip. J Pediatr Orthop B.

[16] Mimura T, Mori K, Kawasaki T, Imai S, Matsusue Y (2014). Triple pelvic osteotomy: Report of our mid-term results and review of literature. World J Orthop.

[17] Fu M, Xiang S, Zhang Z, Huang G, Liu J, Duan X, Yang Z, Wu P, Liao W (2014). The biomechanical differences of rotational acetabular osteotomy. Chiari osteotomy and shelf procedure in developmental dysplasia of hip. BMC Musculoskelet Disord.

[18] Weinstein SL, Mubarak SJ, Wenger DR (2004). Developmental hip dysplasia and dislocation: Part II. Instr Course Lect.

[19] Haidar RK, Jones RS, Vergroesen DA, Evans GA (1996). Simultaneous open reduction and Salter innominate osteotomy for developmental dysplasia of the hip. J Bone Joint Surg (Br).

[20] Galpin RD, Roach JW, Wenger DR, Herring JA, Birch JG (1989). One-stage treatment of congenital dislocation of the hip in older children, including femoral shortening. J Bone Joint Surg Am.

[21] Tonnis D (1990). Surgical treatment of congenital dislocation of the hip. Clin Orthop Relat Res.

[22] Sankar WN, Young CR, Lin AG, Crow SA, Baldwin KD, Moseley CF (2011). Risk factors for failure after open reduction for DDH: a matched cohort analysis. J Pediatr Orthop.

[23] McKay DW (1974). A comparison of the innominate and the pericapsular osteotomy in the treatment of congenital dislocation of the hip. Clin Orthop Relat Res.

[24] Ahmed E, Mohamed AH, Wael H (2013). Surgical treatment of the late - presenting developmental dislocation of the hip after walking age. Acta Ortop Bras.

[25] Severin E (1950). Congenital dislocation of the hip; development of the joint after closed reduction. J Bone Joint Surg Am.

[26] Kalamchi A, MacEwen GD (1980). Avascular necrosis following treatment of congenital dislocation of the hip. J Bone Joint Surg Am.

[27] Braatz F, Eidemuller A, Klotz MC, Beckmann NA, Wolf SI, Dreher T (2014). Hip reconstruction surgery is successful in restoring joint congruity in patients with cerebral palsy: long-term outcome. Int Orthop.

[28] Salter RB, Dubos JP (1974). The first fifteen year’s personal experience with innominate osteotomy in the treatment of congenital dislocation and subluxation of the hip. Clin Orthop Relat Res.

[29] Forlin E, Munhoz da Cunha LA, Figueiredo DC (2006). Treatment of developmental dysplasia of the hip after walking age with open reduction, femoral shortening, and acetabular osteotomy. Orthop Clin North Am.

[30] Ganger R, Radler C, Petje G, Manner HM, Kriegs-Au G, Grill F (2005). Treatment options for developmental dislocation of the hip after walking age. J Pediatr Orthop B.

[31] Kershaw CJ, Ware HE, Pattinson R, Fixsen JA (1993). Revision of failed open reduction of congenital dislocation of the hip. J Bone Joint Surg (Br).

[32] Kamath SU, Bennet GC (2005). Re-dislocation following open reduction for developmental dysplasia of the hip. Int Orthop.

[CR33] The pre-publication history for this paper can be accessed here:http://www.biomedcentral.com/1471-2474/15/401/prepub

