# Supplementary figures and images for: Analyses of outcomes of one-stage operation for treatment of late-diagnosed developmental dislocation of the hip: 864 hips followed for 3.2 to 8.9 years
Source: BMC Musculoskelet Disord. 2014 Nov 28;15:401. doi: 10.1186/1471-2474-15-401 (PMC4289045; doi:10.1186/1471-2474-15-401)

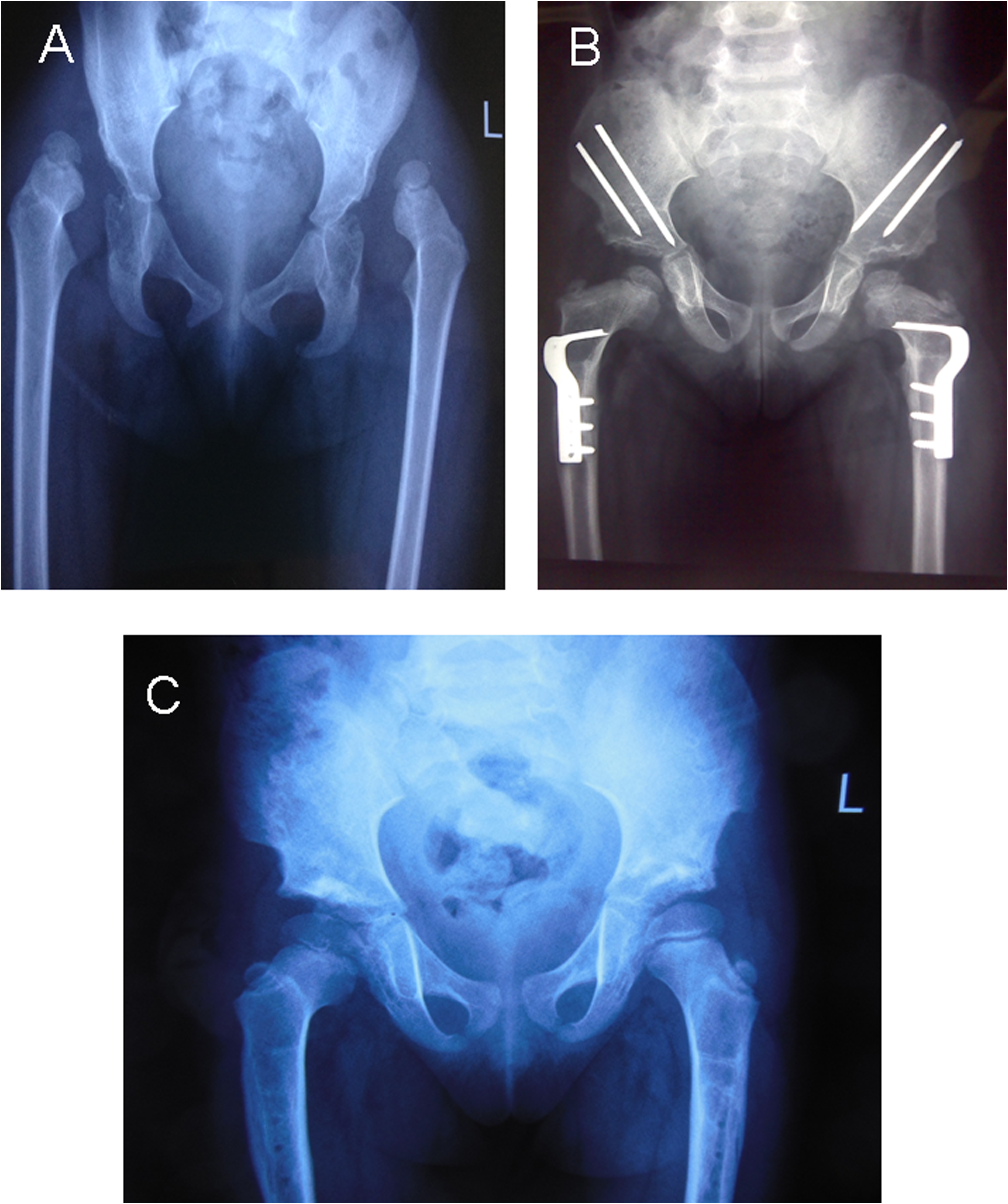

Supplement: Supplementary file 1 — Authors’ original file for figure 1 [file 12891_2014_2371_MOESM1_ESM.tif]

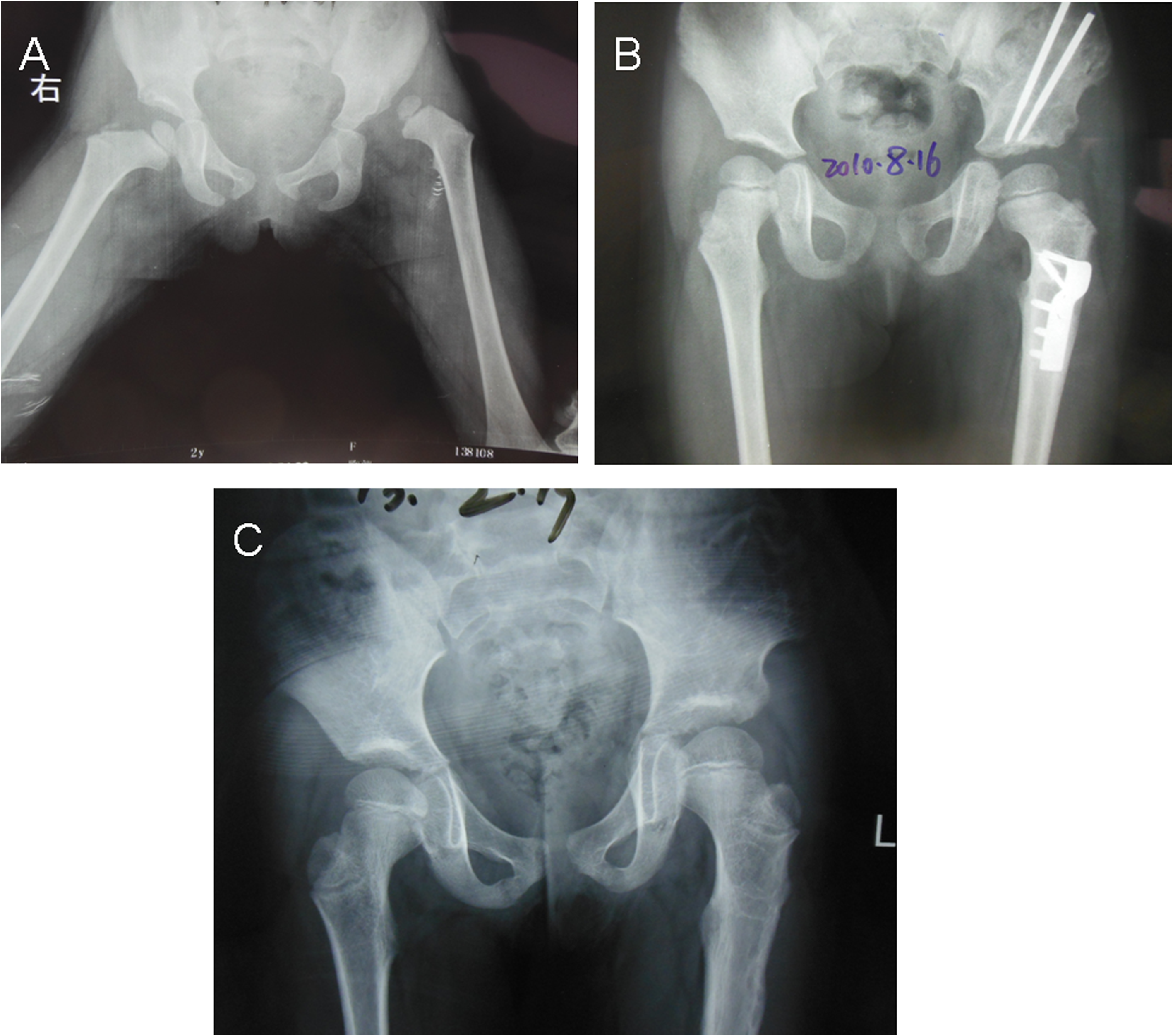

Supplement: Supplementary file 2 — Authors’ original file for figure 2 [file 12891_2014_2371_MOESM2_ESM.tif]
